# EphrinB/EphB forward signaling in Müller cells causes apoptosis of retinal ganglion cells by increasing tumor necrosis factor alpha production in rat experimental glaucomatous model

**DOI:** 10.1186/s40478-018-0618-x

**Published:** 2018-10-24

**Authors:** Shu-Ting Liu, Shu-Min Zhong, Xue-Yan Li, Feng Gao, Fang Li, Meng-Lu Zhang, Ke Zhu, Xing-Huai Sun, Xin Wang, Yanying Miao, Xiong-Li Yang, Zhongfeng Wang

**Affiliations:** 1 Department of Neurology, Institutes of Brain Science, State Key Laboratory of Medical Neurobiology, Zhongshan Hospital, Fudan University, 131 Dongan Road, Shanghai, 200032 China; 2 Department of Ophthalmology at Eye & ENT Hospital, Institutes of Brain Science, State Key Laboratory of Medical Neurobiology, Fudan University, Shanghai, 200031 China

**Keywords:** ephrinB/EphB forward signaling, Retinal Müller cells, TNF-α, NR2B, Glaucoma

## Abstract

It was previously shown that EphB/ephrinB reverse signaling in retinal ganglion cells (RGCs) is activated and involved in RGC apoptosis in a rat chronic ocular hypertension (COH) model. In the present work, we first show that ephrinB/EphB forward signaling was activated in COH retinas, and RGC apoptosis in COH retinas was reduced by PP2, an inhibitor of ephrinB/EphB forward signaling. We further demonstrate that treatment of cultured Müller cells with ephrinB1-Fc, an EphB1 activator, or intravitreal injection of ephrinB1-Fc in normal rats induced an increase in phosphorylated EphB levels in these cells, indicating the activation of ephrinB/EphB forward signaling, similar to those in COH retinas. The ephrinB1-Fc treatment did not induce Müller cell gliosis, as evidenced by unchanged GFAP expression, but significantly up-regulated mRNA and protein levels of tumor necrosis factor-α (TNF-α) in Müller cells, thereby promoting RGC apoptosis. Production of TNF-α induced by the activation of ephrinB/EphB forward signaling was mediated by the NR2B subunit of NMDA receptors, which was followed by a distinct PI3K/Akt/NF-κB signaling pathway, as pharmacological interference of each step of this pathway caused a reduction of TNF-α production, thus attenuating RGC apoptosis. Functional analysis of forward and reverse signaling in such a unique system, in which ephrin and Eph exist respectively in a glial element and a neuronal element, is of theoretical importance. Moreover, our results also raise a possibility that suppression of ephrinB/EphB forward signaling may be a new strategy for ameliorating RGC apoptosis in glaucoma.

## Introduction

Erythropoietin-producing hepatocyte receptor (Eph) is the largest family of transmembrane receptor tyrosine kinases [35, 51]. Eph receptors interacting with their ligands (ephrins, Eph receptor-interacting proteins) that are expressed in the membrane of adjacent cells, initiate forward signaling acting on receptor-expressing cells and reverse signaling acting on ephrin-expressing cells [1, 37] . Both types of signaling play crucial roles in physiological processes, such as developmental axonal guidance, cell migration, synaptic plasticity [32, 38, 48, 49]. And this system is also involved in pathological conditions, such as inflammatory neuropathic pain, hyperalgesic condition [2, 12, 35]. In experimental glaucoma models and patients with glaucoma, the expression of EphB and ephrinB were found to be up-regulated at the optic nerve head (ONH) [17, 19, 20, 55]. Our recent study shows that EphB/ephrinB reverse signaling is activated in retinal ganglion cells (RGCs) in chronic ocular hypertension (COH) rat retina, which contributes to RGC apoptosis through elevating the trafficking of Ca^2+^-impermeable GluA2-containing AMPA receptors [16].

It is noteworthy that the expression of EphB1 and ephrinB2 in COH rats is up-regulated in Müller cells and RGCs, respectively [16]. This result suggests a possibility that there may be an activated ephrinB/EphB forward signaling from RGCs to Müller cells in COH retinas. In glaucoma Müller cells are reactivated, known as gliosis, which may be either protective at an early phase by enhancing the production of neurotrophic factors, or detrimental for RGCs at a later phase by promoting the release of inflammatory cytokines and other cytotoxic substances [6, 18, 24]. We therefore explored how ephrinB/EphB forward signaling may regulate the function of Müller cells.

In the present study we first show that this forward signaling is indeed activated in Müller cells in COH retinas. We then examined whether and how the activation of ephrinB/EphB forward signaling in Müller cells may be involved in RGC apoptosis in COH retinas. We further provide evidence, suggesting that the production of pro-inflammatory cytokines tumor necrosis factor-α (TNF-α) in Müller cells could be increased through a distinct NR2B/PI3K/Akt/NF-κB signaling pathway when this forward signaling was activated, thus contributing to RGC apoptosis.

## Materials and methods

All experiments described in this study were carried out in accordance with the National Institutes of Health (NIH) guidelines for the Care and Use of Laboratory Animals, and were approved by the Institutes of Brain Science at Fudan University. All efforts were made to minimize the number of animals used and their suffering. Male Sprague–Dawley rats (weighing 100–150 g), obtained from SLAC Laboratory Animal Co., Ltd. (Shanghai, China), were housed on a 12 h light/dark schedule.

### Rat COH model

COH rats were produced by injecting the micro-magnetic beads (10 μl, diameter ≈ 10 μm, BioMag®Superparamagnetic Iron Oxide, Bangs Laboratories, Ins) into the anterior chamber of the left eyes following the procedure previously described in detail [11]. Sham-operated treatment, following a similar procedure (except for injecting the same volume of normal saline), was conventionally done on the eyes of other rats. Intraocular pressure (IOP) was measured, using a handheld digital rebound tonometer (TonoLab, Icare, Finland), in the morning to avoid possible circadian differences. The IOPs of both eyes were recorded before surgery (baseline, 0d), immediately after surgery (G0d), and on the 1st, 2nd, 3rd and 4th week after surgery (G1w, G2w, G3w and G4w, respectively).

### Primary retinal Müller cell culture

Primary Müller cell cultures were prepared following the procedures described before [23]. Briefly, retinas isolated from newborn Sprague–Dawley rats (postnatal day 5) were digested with 0.25% trypsin for 15 min at 37 °C, then mechanically dissociated using fire-polished Pasteur pipettes. The cell suspensions were cultured in the Dulbecco’s modified eagle medium (DMEM/F12; Gibco, Life Technologies, Rockville, MD, USA), supplemented with 10% fetal bovine serum (FBS), 100 U/ml penicillin and 100 μg/ml streptomycin in a humidified 5% CO_2_ circumstance at 37 °C. Non-attached cells and microglia cells were removed by blowing with a fire-polished Pasteur pipette. Müller cells of the third-generation cultured for up to 21 days, were used for experiments. All experiments were performed at least in triplicate on three different batches of cultures.

### Treatment of cells

EphrinB1-Fc or IgG-Fc (control) (R&D systems, Minneapolis, MN, USA) was pre-clustered with goat anti-human IgG-Fc (Jackson ImmunoResearch Labs, Wes Grove, PA, USA) for 60 min at room temperature [57]. Cultured Müller cells were treated by ephrinB1-Fc (500 ng/ml) for different periods of time (1~ 24 h). For the inhibitory experiments, inhibitors were added to the medium 30 min before the ephrinB1-Fc treatment. The inhibitors used in this study were as follows: PP2 and RO25–6981 (Tocris, Minneapolis, MN, USA); LY294002 and PDTC (Calbiochem, San Diego, CA, USA).

### Intravitreal injection

The procedure for intravitreal injection refers to our previous studies [16, 33]. EphrinB1-Fc (0.5 μg/μl, 2 μl), XPro1595 (50 μg/μl, 2 μl) (Xencor, Inc., Monrovia, CA, USA), 4-amino-3-(4-chlorophenyl)-1-(*t*-butyl)-1H-pyrazolo [3,4-d] pyrimidine (PP2; 100 μM, 2 μl) or normal saline (2 μl) was injected into the vitreous using a microinjector (Hamilton).

### Real-time PCR

Total RNA was isolated from cultured Müller cells using RNAiso Plus (Takara Co., Japan). Real-time polymerase chain reaction (PCR) was performed as previously described [22]. Forward and reverse primer sequences were 5′-GAG CTG AGC GTG TGT GAC AG-3′ (melting temperature (Tm): 61.9) and 5’-CGC CAG CCA ATT CTC TTT TTG C-3′ (Tm: 60.0) for BDNF, 5’-GCG GTT CCT GTG AAG CGG CCG A-3′ (Tm: 67.5) and 5′-TAG ATA CAT CCA CAC CGT TTA GCG G-3′ (Tm: 61.9) for GDNF, 5’-CTT CAG CAT TCC CTT GAC AC-3′ (Tm: 57.8) and 5’-AGC CTT CCT GCT GAG CAC ACA-3′ for NGF (Tm: 61.9), 5’-AAGTTCCCAAATGGGCTC-3′ (Tm: 55.0) and 5’-TCACAGAGCAATGACTCCAAAG-3′ (Tm: 58.2) for TNF-α, 5’-ATAGCAGCTTTCGACAGTGAG-3′ (Tm: 58.0) and 5’-GTCAACTATGTCCCGACCATT-3′ (Tm: 58.0) for IL-1β, 5’-TTCCCTACTTCACAAGTC-3′ (Tm: 52.7) and 5’-CTAGGTTTGCCGAGTAGA-3′ (Tm: 55.0) for IL-6, 5’-TCA TGA AGT GTG ACG TGG ACA TC-3′ (Tm: 60.2) and 5′-TGT TGC ATT TGC GGG GAC GAT G-3′ (Tm: 61.9) for β-actin, respectively [44, 59]. The thermal cycling conditions were 95 °C for 2 min and 40 cycles of 45 s at 95 °C, 45 s at 58 °C or 60 °C and 45 s at 72 °C. The amplification reaction was performed by using an amplification device (Eppendorf, realplex 4, GER), yielding a melting curve. The data were analyzed using 2^-ΔΔct^ calculation method.

### Western blotting

Western blotting was conducted as previously described [11, 23, 33]. For whole-cell protein extraction, Müller cells were collected and lysed in RIPA buffer supplemented with protease and phosphatase inhibitor cocktail (Roche, supplemented, Mannheim, Germany). For nuclear and cytoplasmic protein extraction, the instructions provided for Nuclear-Cytosol Extraction kit (Applygen Technologies, Inc., Beijing, China) were followed. The concentration of proteins was measured using a standard bicinchoninic acid (BCA) assay kit (Pierce Biotechnology, Rockford, IL, USA). The extracted protein samples were separated on an 8% or 10% SDS-PAGE gel and electrotransferred to PVDF membranes (Immobilon-P, Millipore, Billerica, MA, USA). The following primary antibodies were used: monoclonal mouse anti-β-actin (A5316, 1:10000, Sigma-Aldrich, St. Louis, MO, USA), polyclonal rabbit anti-phosphoY594 EphB1/EphB2 (ab61791, 1:500, Abcam, Cambridge, MA, USA), polyclonal goat anti-EphB1 (AF542, 1:500, R&D systems), monoclonal mouse anti-PI3K p85α (sc-1637, 1:1000; Santa Cruz Biotechnology), polyclonal rabbit anti-PI3K p110 (sc-7189, 1:1000, Santa Cruz Biotechnology), polyclonal rabbit anti- Akt1/2/3 phospho Ser473 (sc-7985-R, 1:1000, Santa Cruz Biotechnology), monoclonal mouse anti-Akt1 (sc-5298, 1:1000, Santa Cruz Biotechnology), polyclonal rabbit anti-NF-κB p65 (sc-372, 1:1000, Santa Cruz Biotechnology), monoclonal mouse anti-NMDAR1 (#556308, 1:500, BD Pharmingen, Franklin Lakes, NJ, USA), polyclonal rabbit anti-NMDA NR2B phospho Tyr1472 (#454583, 1:500, Calbiochem), polyclonal rabbit anti-NMDAR2B (ab65783, 1:500, Abcam), monoclonal mouse anti-GFAP (G6171, 1:500, Sigma-Aldrich). The membranes were incubated with Donkey anti-mouse, rabbit or goat IgG HRP (Jackson ImmunoResearch Labs) for 1.5 h at room temperature, and then incubated with enhanced chemofluorescent reagent (Pierce Biotechnology). The blots were imaged with a digital imager (FluorChem E System, ProteinSimple, USA) and protein bands were quantitatively analyzed with Alpha View software (Cell Biosciences, Inc.).

### Immunofluorescent staining

Immunofluorescent staining was performed following the procedure previously described [43, 70]. Briefly, cultured Müller cells, grown on cover slips, were fixed with 4% paraformaldehyde (PFA) for 20 min. For rats with the intravitreal injection, retinas were fixed with 4% PFA for 2 h and dehydrated with graded sucrose solutions at 4 °C (2 h in 10%, 2 h in 20% and overnight in 30%), and then vertically sectioned at a thickness of 14 μm (Leica, Nussloch, Germany), mounted on chrome-alum-gelatin-coated slides (Fisher Scientific, Pittsburgh, PA, USA). After washing in PBS, the cultured cells or retinal sections were blocked for 1.5 h in 10% donkey serum, 3% BSA and 0.1% Triton X-100, and then incubated with the following primary antibodies at 4 °C overnight: monoclonal mouse anti-GFAP (G6171; 1:400; Sigma-Aldrich), polyclonal rabbit anti-NF κB p65 (sc-372, 1:200, Santa Cruz Biotechnology). Secondary antibodies including 488-conjugated donkey anti-mouse or rabbit IgG (1:800 dilution) (Jackson ImmunoResearch Labs) were used. After washing, the cover slips or sections were mounted with anti-fade mounting medium with DAPI (Vector Laboratories, Burlingame, CA, USA) and photographed with a Leica SP2 confocal laser-scanning microscope.

### Enzyme-linked immunoabsorbent assay

The concentration of TNF-α protein was measured by enzyme-linked immunoabsorbent assay (ELISA). The supernatants of cultured Müller cells were collected and centrifuged. For rats with the intravitreal injection, retinas were collected at day 3 (3 d) and day 7 (7 d) after the injection and lysed in RIPA buffer. ELISA were carried out with anti-rat-TNF-α ELISA kits (R&D Systems), following the manufacturer instructions.

### Assay of cell apoptosis

To detect cell apoptosis, terminal dUTP nick end labeling (TUNEL) assay [10, 16] was performed on whole flat-mounted retinas following the manufacturer instructions (Promega, Madison, WI, USA). TUNEL positive signals were visualized with a confocal laser scanning microscope through a 10× objective (FluoView 1000, Olympus), and all these that merged well with DAPI in each retina were counted.

### Data analysis

All experiments on cultured cells were performed at least in triplicate on three different batches of cultures. The detailed animal number used in each experiment was given in the “Figure legends”. All data were presented as means ± S.E.M., and analyzed using GraphPad Prism software V 6.02 (GraphPad software, Inc., La Jolla, CA, USA). One-way ANOVA with Tukey’s post-hoc test (multiple comparisons) or two-way ANOVA with Bonferroni’s multiple comparisons test were used as appropriate. Statistical results were considered significant if *P* value is less than 0.05.

## Results

### EphrinB/EphB forward signaling is activated in COH retinas

**Fig. 1:**
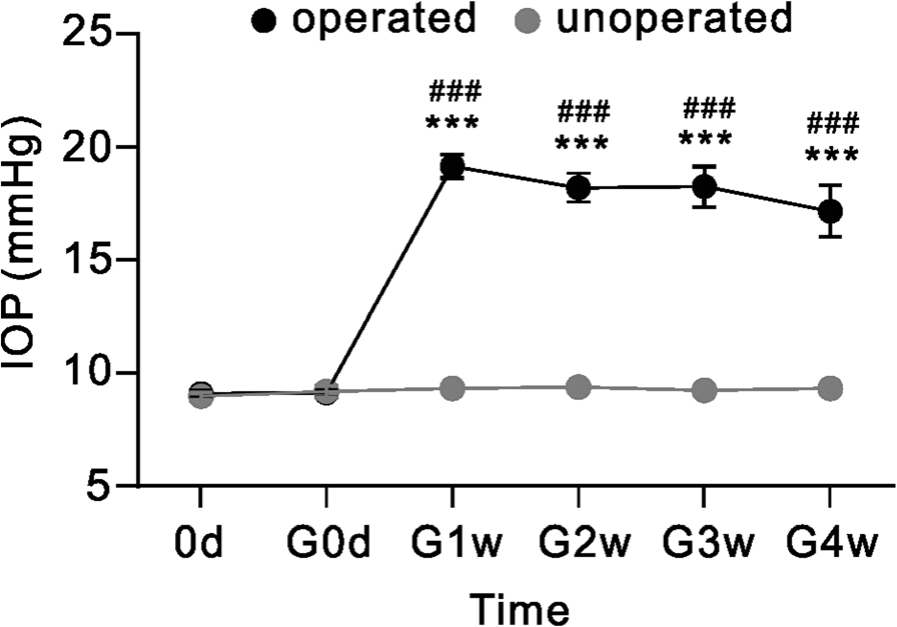
Changes of IOP of both eyes in COH rats. IOP elevation after the injection of micro-magnetic beads in left eyes (operated eyes) as a function of time. ***P < 0.001 vs. 0d, and ^###^*P* < 0.001 vs. unoperated eyes (right eyes) at the same time point

**Fig. 2:**
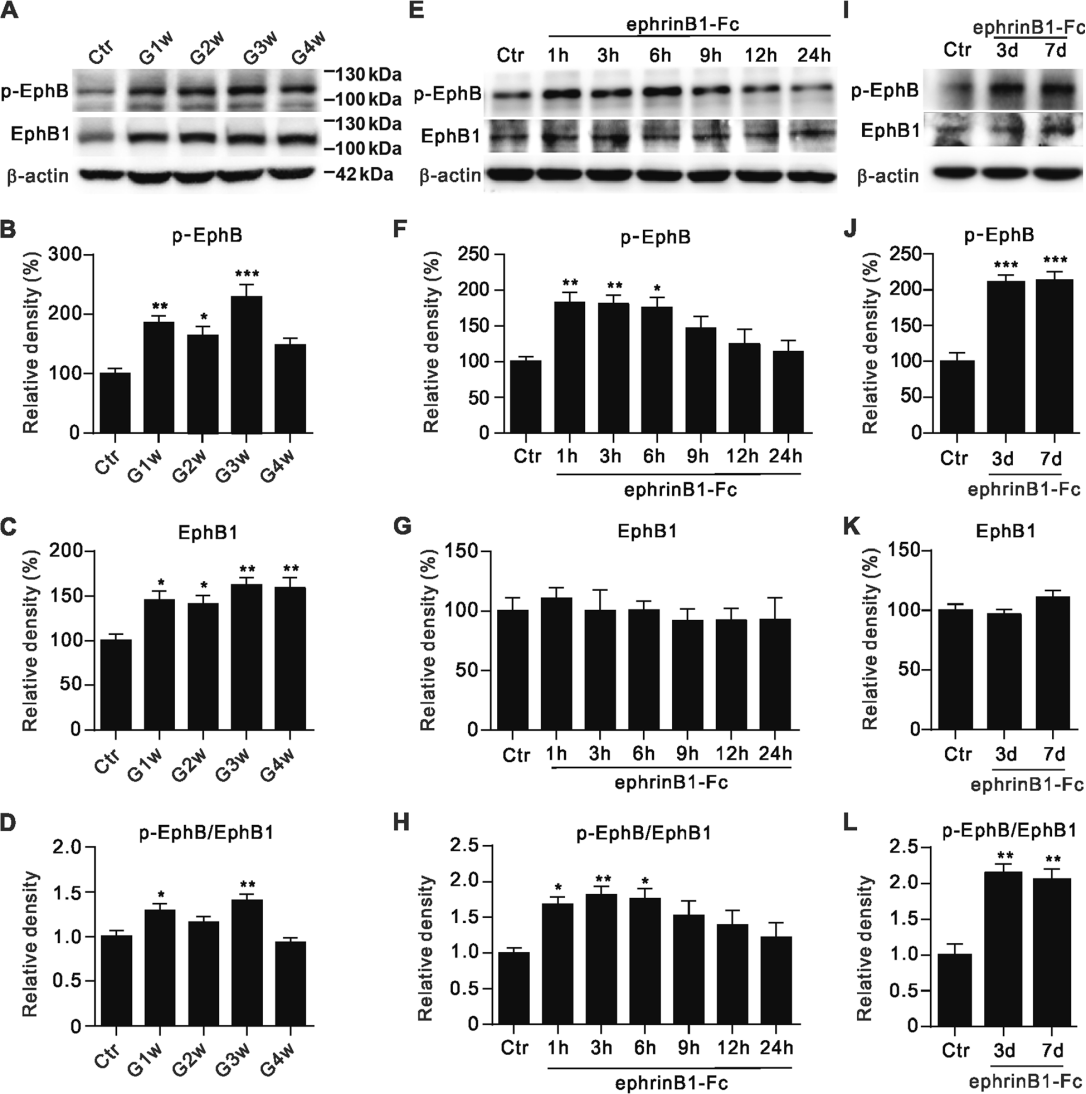
EphrinB/EphB forward signaling is activated in retinal Müller cells by IOP elevation and ephrinB1-Fc treatment. **a**, Representative immunoblots showing the changes in EphB1 and phosphorylated EphB (p-EphB) expression in sham-operated (control, Ctr) and COH retinal extracts from G1w to G4w. **b-d**, Bar charts summarizing the average densitometric quantification of immunoreactive bands of p-EphB (**b**), EphB1 (**c**) and the average p-EphB/EphB1 ratios (**d**) obtained in COH retinal extracts from G1w to G4w. *n* = 5 for all groups. **e**, Representative immunoblots showing the changes in EphB1 and p-EphB levels in Müller cell extracts treated with IgG-Fc (500 ng/ml) (Ctr) and those treated with ephrinB1-Fc (500 ng/ml) for different periods of time (1–24 h). **f-h**, Bar charts summarizing the average densitometric quantification of immunoreactive bands of p-EphB (**f**), EphB1 (**g**) and the average p-EphB/EphB1 ratios (**h**) in Müller cells treated with ephrinB1-Fc for different periods of time, as compared to those in control condition. n = 5 for all groups. **i**, Representative immunoblots showing the changes in EphB1 and p-EphB expression in normal saline-injected retina (Ctr), and ephrinB1-Fc-injected retinas (0.5 μg/μl, 2 μl) at different post-injection times. **j-l**, Bar charts summarizing the average densitometric quantification of immunoreactive bands of p-EphB (**j**), EphB1 (**k**) and the average ratios of p-EphB/EphB1 (**l**) in retinal extracts under control condition and those of ephrinB1-Fc-injected retinas at different post-injection times (3 d and 7 d). *n* = 4 for all groups. All the data are normalized to their corresponding β-actin and then to Ctr. * *P* < 0.05, ** *P* < 0.01 and *** *P* < 0.001 vs. Ctr

As shown in Fig. 1, in COH rats the average IOPs of operated eyes (left eyes) were kept at high levels from G1w to G4w (19.2 ± 0.5 mmHg to 17.2 ± 1.1 mmHg, *n* = 6~ 24), which was significantly higher than that at 0d (9.1 ± 0.2 mmHg, *n* = 24), and of those of unoperated eyes (right eyes) (9.1 ± 0.2 mmHg to 9.3 ± 0.2 mmHg, n = 6~ 24) (*P* all < 0.001). The average IOPs of sham-operated eyes (control) was 9.1 ± 0.2 mmHg (*n* = 18), which was not significantly different from the value in COH rats at 0d (*P* > 0.05). To find out whether ephrinB/EphB forward signaling was activated in rat COH retinas, EphB1 and p-EphB protein levels of retinal extracts obtained from COH rats were determined at different post-operational times by Western blotting. Just like reported previously [16], EphB1 protein levels were significantly increased to 145.2 ± 10.6% of that in sham-operated group (control) (*n* = 5, *P* = 0.020 vs. control) on G1w post-operationally and then remained at this higher level throughout G4w (Fig. 2a, c). Moreover, the phosphorylated EphB (p-EphB) level, which is regarded as a sign of EphB1 activation, was also increased from G1w to G3w, thus resulting in increased p-EphB/EphB ratios on G1w and G3w (Fig. 2b, d), and then returned to the control level.

The changes in both EphB1 and p-EphB in COH retinas may be complicated by the possible involvement of Müller cell gliosis, which is induced in COH retinas. To determine activation of ephrinB/EphB forward signaling did occur in Müller cells, we examined how administration of ephrinB1-Fc, an activator of EphB, change EphB and p-EphB levels in purified Müller cells and in intact normal retinas. Fig. 2e shows the EphB1 and p-EphB protein levels when purified cultured Müller cells were treated with ephrinB1-Fc (500 ng/ml) for different periods of time. While total EphB1 protein levels showed no significant changes over a 24 h period, p-EphB levels were clearly increased at 1 h and remained at the higher level thereafter until 6 h (Fig. 2e-f). The ratio of p-EphB/EphB1 started increasing at 1 h following ephrinB1-Fc treatment, peaking at 3 h, and then fluctuated around this higher level until 6 h, which was followed by a decline to the control level (Fig. 2h).

To facilitate the analysis of roles of ephrinB/EphB forward signaling activation and possible underlying signaling pathways, we also examined effects of ephrinB1-Fc on EphB and p-EphB levels in intact normal retinas. Like in COH retinas, intravitreal injection of ephrinB1-Fc (0.5 μg/μl, 2 μl) significantly increased the p-EphB level in retinal extracts, but did not much alter the EphB1 level (Fig. 2i-l).

**Fig. 3:**
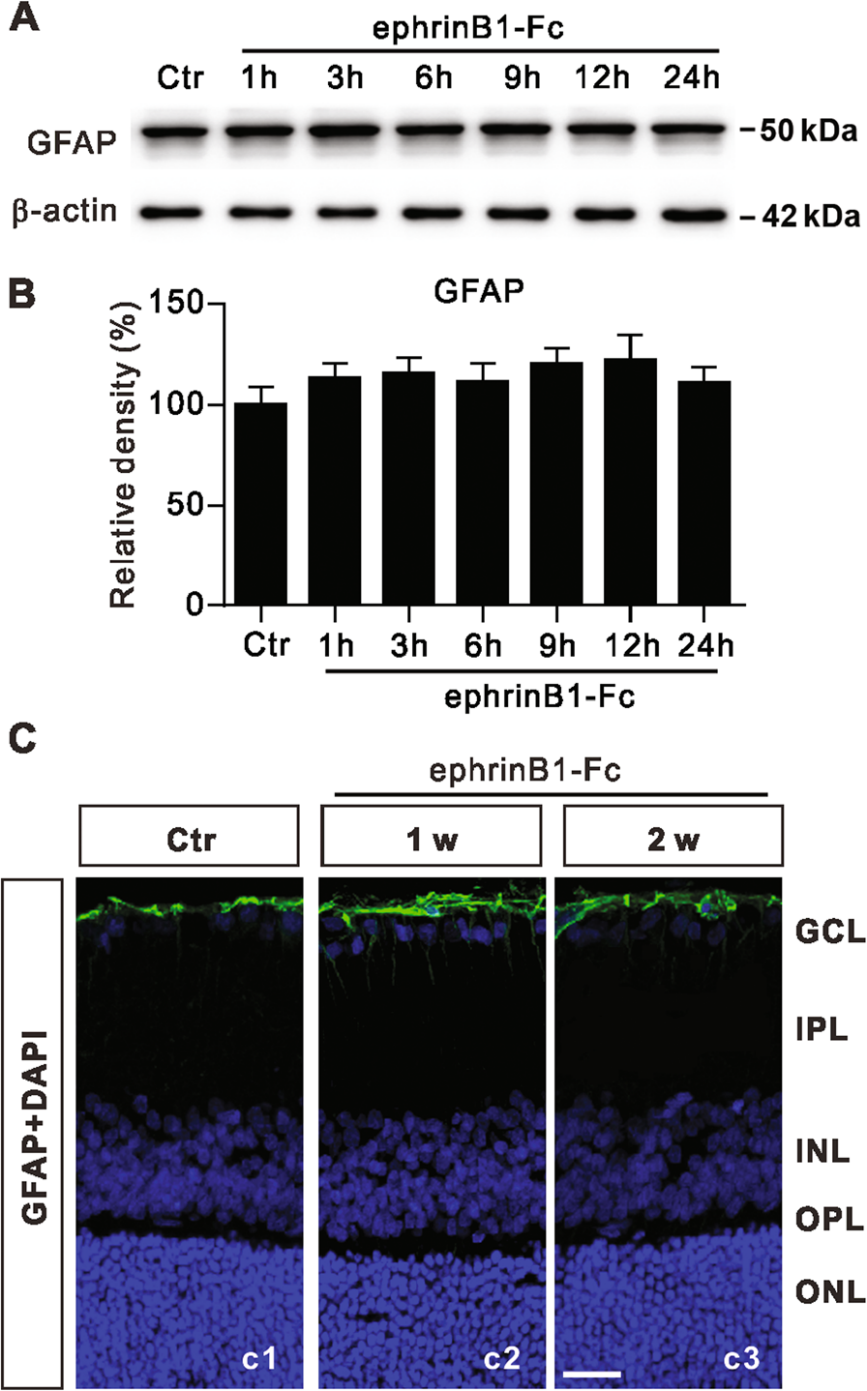
Changes in GFAP protein levels in ephrinB1-Fc treated Müller cells and ephrinB1-Fc injected retinas. **a**, Representative immunoblots showing the changes in GFAP protein levels in Müller cell extracts treated with IgG-Fc (500 ng/ml) (Ctr) and those treated with ephrinB1-Fc (500 ng/ml) for different periods of time (1–24 h). **b**, Bar charts summarizing the average densitometric quantification of immunoreactive bands of GFAP under the conditions as shown in *A*. *n* = 6 for all groups. **c**, Immunofluorescence labeling showing GFAP expression in rat retinal vertical slices taken from normal saline-injected retina (c1) (Ctr) and ephrinB1-Fc-injected retinas on 1 and 2 weeks (c2 and c3) after the injection. Scale bar, 20 μm, for all the images. GCL, ganglion cell layer; IPL, inner plexiform layer; INL, inner nuclear layer; OPL, outer plexiform layer; ONL, outer nuclear layer

To test whether activated ephrinB/EphB forward signaling may induce Müller cell gliosis, GFAP protein levels were determined for cultured Müller cells treated with ephrinB1-Fc for different periods of time. As shown in Fig. 3a-b, GFAP protein expression was not significantly changed following ephrinB1-Fc treatment for different periods of time. Furthermore, expression of GFAP in Müller cells was also examined in normal retinas treated with intravitreal injection of ephrinB1-Fc and collected on 1 w and 2 w after the injection. GFAP labeling in the retina was hardly changed by ephrinB1-Fc injection (Fig. 3c). In both ephrinB1-Fc and normal saline-injected (control) sections GFAP labeling was strictly limited to the endfeet of Müller cells. These results suggest that activation of ephrinB/EphB forward signaling does not induce Müller cell gliosis.

### Activation of ephrinB/EphB forward signaling is involved in RGC apoptosis

**Fig. 4:**
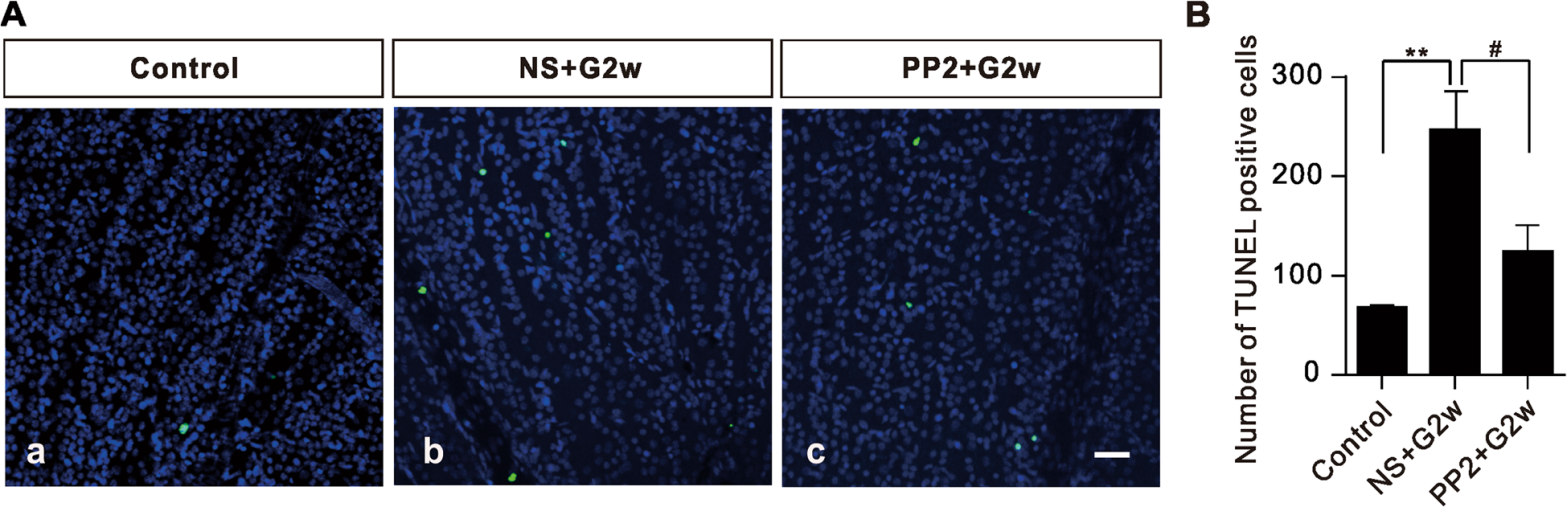
Inhibition of ephrinB/EphB forward signaling reduces RGC apoptosis. **a**, Representative TUNEL staining images of RGC apoptosis in sham (control) (a), normal saline (NS)-injected (NS + G2w) (b), and PP2-injected (PP2 + G2w) (c) whole flat-mounted retinas on G2w in the regions at angle 0°. Scale bar, 50 μm, for all the images. **b**, Bar chart showing the average numbers of TUNEL-positive cells in whole flat-mounted retinas under different conditions. *n* = 4~ 5. **P < 0.01 vs. control; ^#^*P* < 0.05 vs. NS + G2w group

Whether activation of ephrinB/EphB forward signaling is involved in RGC apoptosis in COH retinas was addressed by monitoring changes in TUNEL-positive signals in COH retinas when ephrinB/EphB forward signaling was blocked by intravitreal injection of PP2, an inhibitor of src family tyrosine kinases. As shown in Fig. 4, the TUNEL signals in the COH retinas obtained on G2w were much more than those in the control retinas. It is immediately evident by comparing Fig. 4 Ac and Aa that addition of PP2 made 3 d before the COH operation reduced TUNEL signals dramatically. Statistical comparison of the data is shown in Fig. 4b. It should be noted that PP2 only partially reduced the number of TUNEL-positive signals in COH retinas, suggesting that some factor(s) other than the activation of ephrinB/EphB forward signaling may contribute to RGC apoptosis in COH retinas.

### Activation of ephrinB/EphB forward signaling induces TNF-α production

**Fig. 5:**
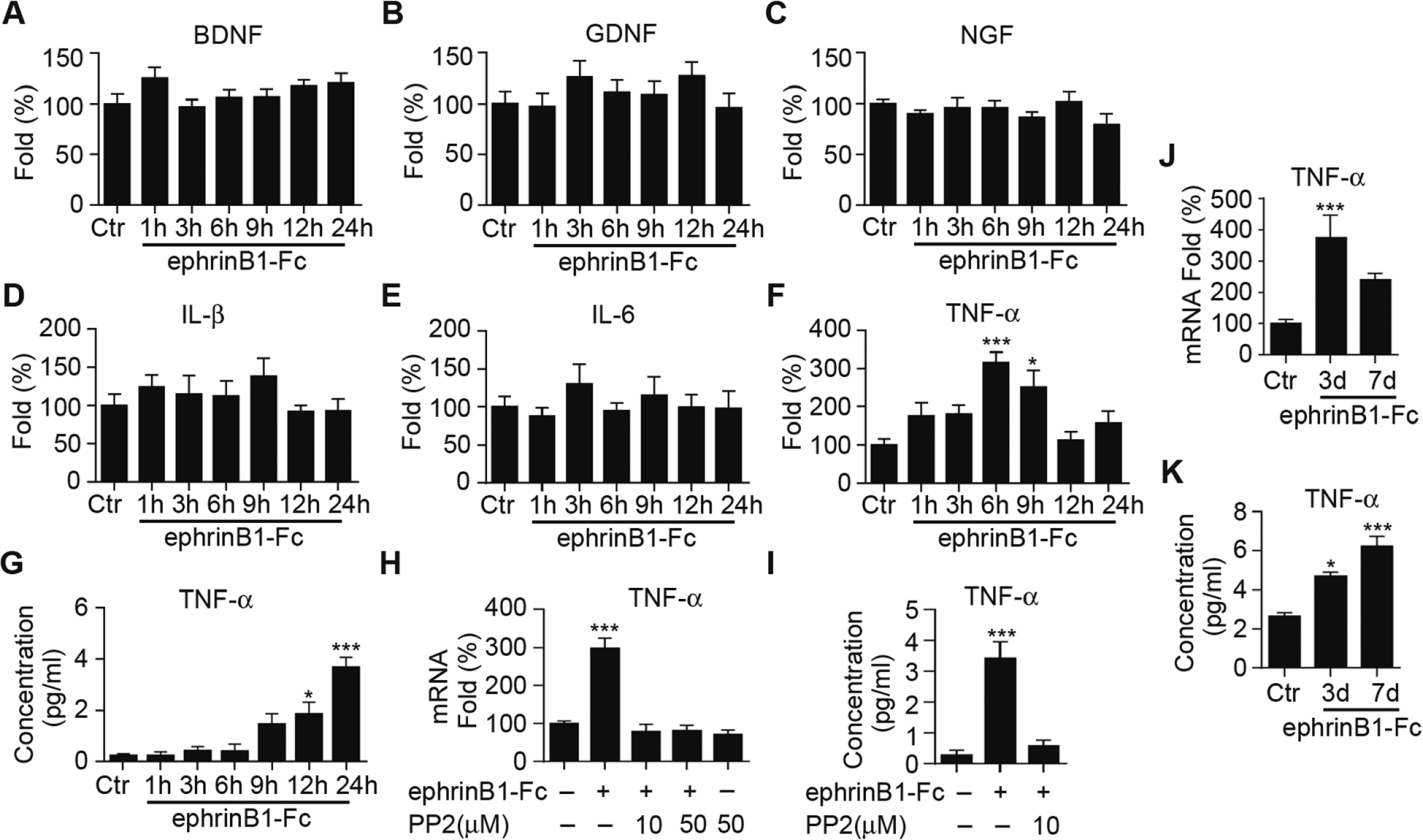
Activation of ephrinB/EphB forward signaling increases TNF-α production in cultured Müller cells and retinal tissues. The cells were treated by IgG-Fc (500 ng/ml) (Ctr) or ephrinB1-Fc (500 ng/ml) for different periods of time (1–24 h). **a-c**, Cumulative data summarizing the changes in mRNA levels of BDNF (**a**), GDNF (**b**) and NGF (**c**) in Müller cell extracts obtained in Ctr and those with ephrinB1-Fc-treatment for different periods of time. *n* = 6 for all groups. **d-f**, Cumulative data summarizing the changes in mRNA levels of IL-1β (**d**), IL-6 (**e**) and TNF-α (**f**) in Müller cell extracts obtained in Ctr and those with ephrinB1-Fc-treatment for different periods of time. The relative abundance of mRNA was quantified using 2^-ΔΔct^ calculation method and expressed as fold change. n = 6 for all groups.* *P* < 0.05 and *** *P* < 0.001 vs. Ctr. **g**, Bar chart showing the average extracellular TNF-α concentrations in IgG-Fc-treated group (Ctr) and groups of ephrinB1-Fc-treatment for different periods of time. n = 6 for all groups. * *P* < 0.05 and *** *P* < 0.001 vs. Ctr. **h**, Summary data showing that the ephrinB1-Fc-treatment induced increase in TNF-α mRNA levels in cultured Müller cells was blocked by pre-incubation with PP2. PP2 (10 μM/50 μM) was added to the medium 30 min before a 6 h ephrinB1-Fc treatment. n = 6 for all groups. *** *P* < 0.001 vs. IgG-Fc treated group (Ctr). **i**, Bar chart showing that pre-incubation with PP2 inhibited the ephrinB1-Fc-treatment induced increase in TNF-α protein levels in cultured Müller cells. PP2 (10 μM) was added to the medium 30 min before a 24 h ephrinB1-Fc treatment. n = 6 for all groups. *** *P* < 0.001 vs. IgG-Fc treated group (Ctr). **j**, Cumulative data summarizing the changes in TNF-α mRNA levels in normal saline-injected retinas (Ctr) and retinas with ephrinB1-Fc-injection at different post-injection times. EphrinB1-Fc (0.5 μg/μl, 2 μl) was intravitreally injected and retinas were collected at different post-injection times (3 d and 7 d). n = 6 for all groups. **k**, Bar chart showing the changes in TNF-α protein levels in Ctr retinas and ephrinB1-Fc injected retinas at different post-injection times (3 d and 7 d). *n* = 4 for all groups. * *P* < 0.05 and *** *P* < 0.001 vs. Ctr

In glaucomatous retinas, activated Müller cells may be either neuroprotective or detrimental for RGCs by releasing neurotrophic factors or cytotoxic substances. Since activation of ephrinB/EphB forward signaling did not induce Müller cell gliosis, what would be the effect of activated ephrinB/EphB forward signaling on Müller cells? To address this question, we examined changes in mRNA levels of neurotrophic factors (BDNF, GDNF and NGF) and inflammatory factors (IL-1β, IL-6 and TNF-α) in cultured Müller cells following ephrinB1-Fc treatment, using real-time PCR technique. As shown in Fig. 5a-e, the mRNA levels of BDNF, GDNF, NGF, IL-1β and IL-6 all fluctuated around the control level, exhibiting no significant changes with time following ephrinB1-Fc treatment. In contrast, the mRNA level of TNF-α was increased at 6 h and 9 h respectively after ephrinB1-Fc treatment (Fig. 5f). It should be noted that the TNF-α mRNA level declined quickly and dramatically at 9 h after the ephrinB1-Fc treatment so that the values obtained at 12 h and 24 h were not different from the control one.

TNF-α protein concentrations in the culture medium of Müller cells were also evaluated as a function of time after ephrinB1-Fc treatment by ELISA assay. Almost no TNF-α protein could be detected in the control group (IgG-Fc) (Fig. 5g). In the ephrinB1-Fc-treated group, TNF-α proteins were also hardly detectable in the first 6 h, but were steadily elevated at 9 h and thereafter (at 12 h and 24 h). The effects of ephrinB1-Fc on TNF-α mRNA and protein levels were not seen in Müller cells pre-incubated with PP2. Figure 5h shows that in these cells pretreated with PP2 (10 μM or 50 μM) for 30 min beforehand, ephrinB1-Fc no longer increased the TNF-α mRNA level at 6 h. Similarly, the increase in TNF-α protein concentration in the culture medium of Müller cells treated with ephrinB1-Fc for 24 h was also blocked by pre-incubation of PP2 (Fig. 5i).

Intravitreal injections of ephrinB1-Fc also elevated TNF-α mRNA and protein levels in intact normal retinas. In these experiments, animals treated with normal saline constituted a control group. The TNF-α mRNA level was significantly increased to 384.1 ± 74.5% of control at 3 d. The level became lower at 7 d, but still higher than the control level (Fig. 5j). Similarly, the protein concentration of TNF-α in the ephrinB1-Fc injected retinas was steadily increased to at 3 d and 7 d (Fig. 5k). These results suggest that activation of ephrinB/EphB forward signaling did induce an increase in TNF-α synthesis and release in Müller cells.

**Fig. 6:**
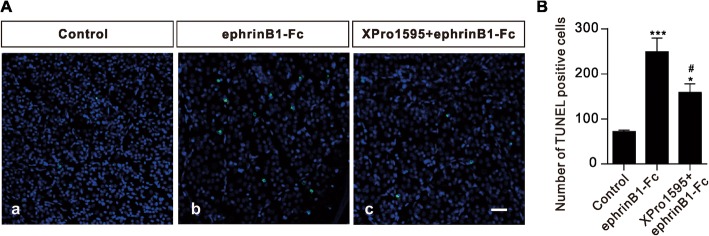
Inhibition of TNF-α reduces RGC apoptosis in ephrinB1-Fc-injected retinas. **a**, Representative TUNEL staining images of RGC apoptosis in normal saline-injected (control) (a), ephrinB1-Fc-injected (b), and ephrinB1-Fc plus Xpro1595-injected (c) whole flat-mounted retinas on 7 d after the injections in the regions at angle 0°. Scale bar, 50 μm, for all the images. **b**, Bar chart showing the average number of TUNEL-positive cells in whole flat-mounted retinas under different conditions. XPro1595 (50 μg/μl, 2 μl) was pre-injected 1 d before the ephrinB1-Fc injection. *n* = 5 for all groups. *P < 0.05 and ***P < 0.001 vs. control; ^#^*P* < 0.05 vs. ephrinB1-Fc alone group

When ephrinB/EphB forward signaling activation-induced TNF-α production from Müller cells was suppressed, RGC apoptosis was largely reduced. Figure 6 shows that numerous TUNEL-positive signals were seen in the ephrinB1-Fc injected retina (Fig. 6Ab), much more than those detected in the normal saline injected control retina (Fig. 6Aa). Furthermore, intravitreal co-injection of XPro1595, a selective inhibitor of soluble TNF-α [3], remarkably reduced the number of TUNEL-positive signals in the ephrinB1-Fc injected retina (Fig. 6a-b), meaning a reduction of the ephrinB1-Fc induced effect. The fact that the number was still higher than the control one suggests that only part of RGC apoptosis induced by activation of ephrinB/EphB forward signaling may be mediated by increased production of TNF-α from Müller cells.

### NR2B/PI3K/Akt/NF-κB signaling pathway mediates TNF-α production induced by ephrinB/EphB forward signaling activation

**Fig. 7:**
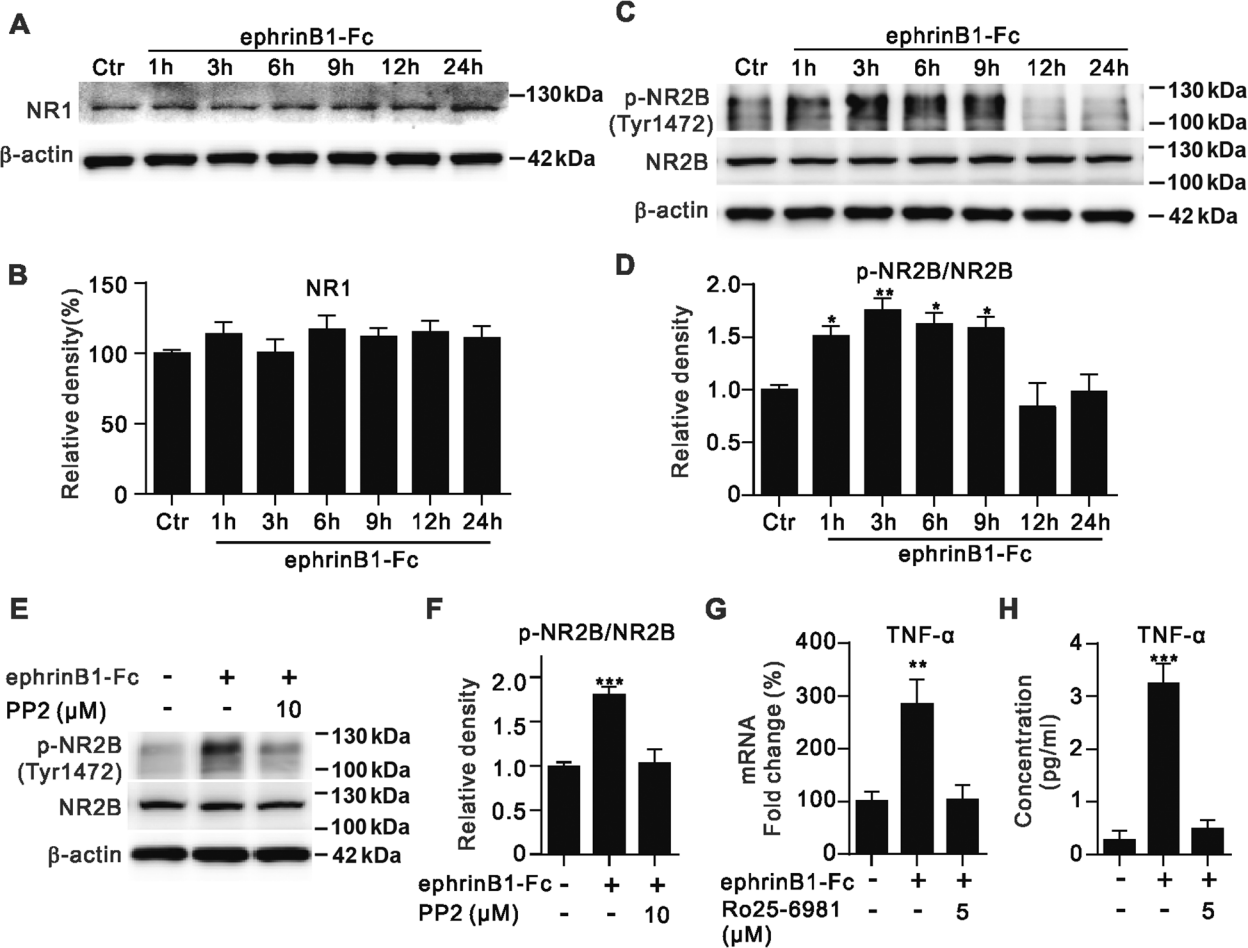
EphrinB1-Fc treatment induces an increase in p-NR2B protein levels and increases TNF-α mRNA and protein levels in cultured Müller cells. **a**, Representative immunoblots showing the protein levels of NR1 in IgG-Fc-treated cells (Ctr), and those treated with ephrinB1-Fc for different periods of time. **b**, Bar chart summarizing the average densitometric quantification of immunoreactive bands of NR1. n = 4 for all groups.**c**, Representative immunoblots showing the protein levels of phosphorylated NR2B (p-NR2B) and NR2B in Müller cells treated with ephrinB1-Fc for different periods of time. **d**, Bar chart summarizing the average p-NR2B/NR2B ratios in Müller cells treated with ephrinB1-Fc for different periods of time. n = 6~ 7. * *P* < 0.05 and ** *P* < 0.01 vs. Ctr. **e**, Representative immunoblots showing that pre-incubation with PP2 eliminated the ephrinB1-Fc-treatment induced changes in p-NR2B protein levels in Müller cells. PP2 (10 μM) was added to the medium 30 min before a 3 h ephrinB1-Fc-treatment. **f**, Bar charts summarizing the average p-NR2B/NR2B ratios obtained in the presence of ephrinB1-Fc, with or without 30 min pre-incubation of 10 μM PP2. All the data are normalized to the ratio obtained in IgG-Fc treated group (Ctr). n = 4 for all groups. *** *P* < 0.001 vs. IgG-Fc treated group (Ctr). **g**, Summary data showing that relative TNF-α mRNA levels in Müller cells, represented as fold changes, were significantly elevated by ephrinB1-Fc treatment, but the elevation was abolished by addition of RO25–6981 (5 μM) 30 min before the ephrinB1-Fc treatment. *n* = 5 for all groups. ** *P* < 0.01 vs. Ctr (IgG-Fc treated group). **h**, Summary data showing that the ephrinB1-Fc treatment induced increase in TNF-α protein levels of cultured Müller cells was blocked by adding RO25–6981 (5 μM) to the medium 30 min before the ephrinB1-Fc treatment. n = 5 for all groups. *** *P* < 0.001 vs. Ctr (IgG-Fc treated group)

To understand how activated Ephrin/Eph forward signaling causes the production of TNF-α from Müller cells, possible involvement of NMDA receptors was examined. It was reported that in excitotoxic loss of retinal neurons in mice TNF-α protein production in Müller cells plays a crucial role, which was mediated through NMDA receptor-induced intracellular signaling mechanisms [40]. Moreover, the NMDA receptor antagonist memantine displays protective effects against streptozotocin treatment-induced activation of rat astrocytoma cell line by reducing TNF-α protein levels [54]. Since NMDA receptor subunits, such as NR1 and NR2A-D, are expressed in Müller cells [39, 53], we therefore tested changes in expression of NR1 and NR2B subunits in cultured Müller cells following ephrinB1-Fc treatment. These two subunits were chosen because there is evidence that the ephrinB/EphB system could directly interact with NR1 and modulate tyrosine phosphorylation of NR2B [14, 25, 56, 60]_._ EphrinB1-Fc treatment for different periods of time did not significantly affect protein levels of either NR1 or NR2B (Fig. 7a-c) in cultured Müller cells. However, the levels of phosphorylated NR2B at Tyr1472 site (p-NR2B) were steadily increased over time (Fig. 7c-d) so that the ratio p-NR2B/NR2B was increased at 1 h, and remained around this higher level throughout 9 h. The ratio tended to decline to the control level thereafter. This elevation of p-NR2B protein was prevented by PP2 pre-incubation (Fig. 7e-f).

Moreover, the increased mRNA and protein levels of TNF-α in Müller cells induced by ephrinB1-Fc treatment could be abolished by 5 μM RO25–6981, a specific NR2B antagonist [29]. As shown in Fig. 7g-h, when the cells were pre-incubated with RO25–6981, both mRNA and protein levels were almost unchanged. These results strongly suggest that the activation of NMDA receptors may be a key step in the signaling pathway mediating ephrinB/EphB forward signaling induced production of TNF-α.

**Fig. 8:**
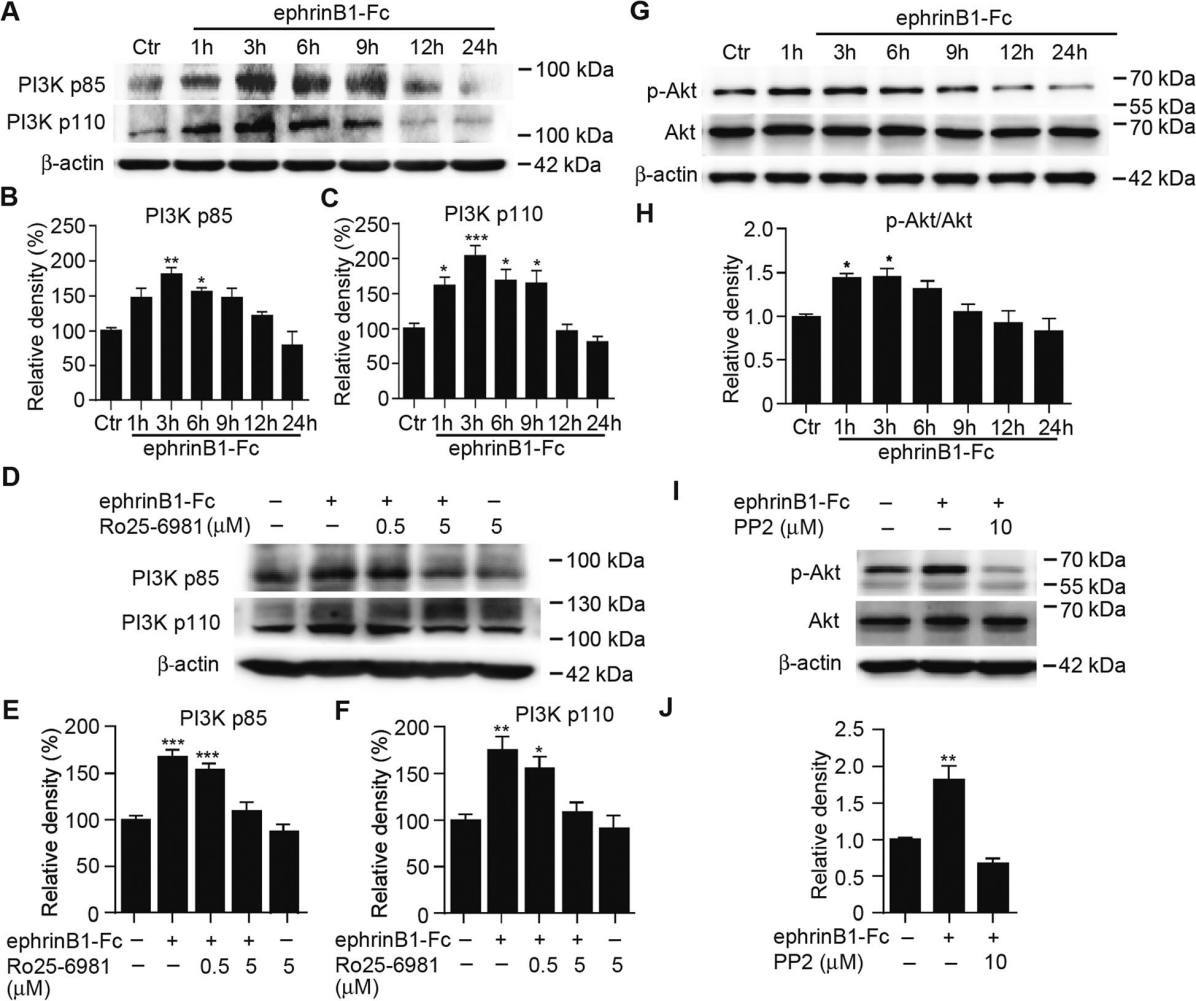
EphrinB1-Fc treatment activates PI3K/Akt signaling in cultured Müller cells. **a**, Representative immunoblots showing the changes in PI3K p85 and p110 protein levels in IgG-Fc-treated cells (Ctr), and those treated with ephrinB1-Fc for different periods of time. **b-c**, Bar chart summarizing the average densitometric quantification of immunoreactive bands of PI3K p85 (**b**) and PI3K p110 (**c**) under different conditions as shown in *A*. *n* = 4 for all groups. All the data are normalized to Ctr. * *P* < 0.05, ** *P* < 0.01 and *** *P* < 0.001 vs. Ctr. **d**, Representative immunoblots showing that pre-incubation with RO25–6981 eliminated the ephrinB1-Fc-treatment induced changes in PI3K p85 and p110 protein levels in Müller cells. RO25–6981 (0.5 μM/5 μM) was added to the medium 30 min before a 6 h ephrinB1-Fc treatment. n = 5 for all groups. **e-f**, Bar chart summarizing the average densitometric quantification of immunoreactive bands of PI3K p85 (**e**) and p110 (**f**) under different conditions as shown in *D*. n = 5 for all groups. *P < 0.05, ** *P* < 0.01 and *** *P* < 0.001 vs. Ctr (IgG-Fc treated group). **g**, Representative immunoblots showing the changes in p-Akt and Akt protein levels in IgG-Fc-treated cells (Ctr), and those treated with ephrinB1-Fc for different periods of time. **h**, Bar chart summarizing the average p-Akt/Akt ratios in Müller cells treated with ephrinB1-Fc for different periods of time. n = 5 for all groups. * *P* < 0.05, vs. Ctr. **i**, Representative immunoblots showing that ephrinB1-Fc treatment induced changes in p-Akt and Akt protein levels were blocked by adding PP2 (10 μM) to the medium 30 min before a 3 h ephrinB1-Fc treatment. **j**, Bar chart summarizing the average p-Akt/Akt ratios of Müller cells under different conditions as shown in *I*. *n* = 5 for all groups. ** *P* < 0.01 vs. Ctr (IgG-Fc treated group)

Phosphatidylinositol 3-kinases (PI3K) are heterodimers composed of a catalytic subunit (p110) and an adaptor/regulatory subunit (p85). Since Ca^2+^ influx through NMDA receptors is known to activate PI3K [4, 9, 34], how this protein level in Müller cells was changed by ephrinB1-Fc treatment was examined. As shown in Fig. 8a-c, the protein levels of PI3K p85 subunit started increasing at 3 h, and slightly declined at 6 h after ephrinB1-Fc treatment, and then returned to the control level. Similar results were obtained for PI3K p110 subunit. The elevation in both p85 and p110 was blocked by pre-incubation with RO25–6981 (Fig. 8d-f).

Akt is a downstream molecule of PI3K, and there is evidence that PI3K/Akt signaling is involved in EphB-mediated events [27, 58, 69]. Western blotting shows that the p-Akt/Akt ratio was increased at 1 h and 3 h, respectively, and declined to the control level during the remaining 12 h (Fig. 8g-h). Again, the increased ratios were abolished by pre-incubation with PP2 (Fig. 8i-j).

**Fig. 9:**
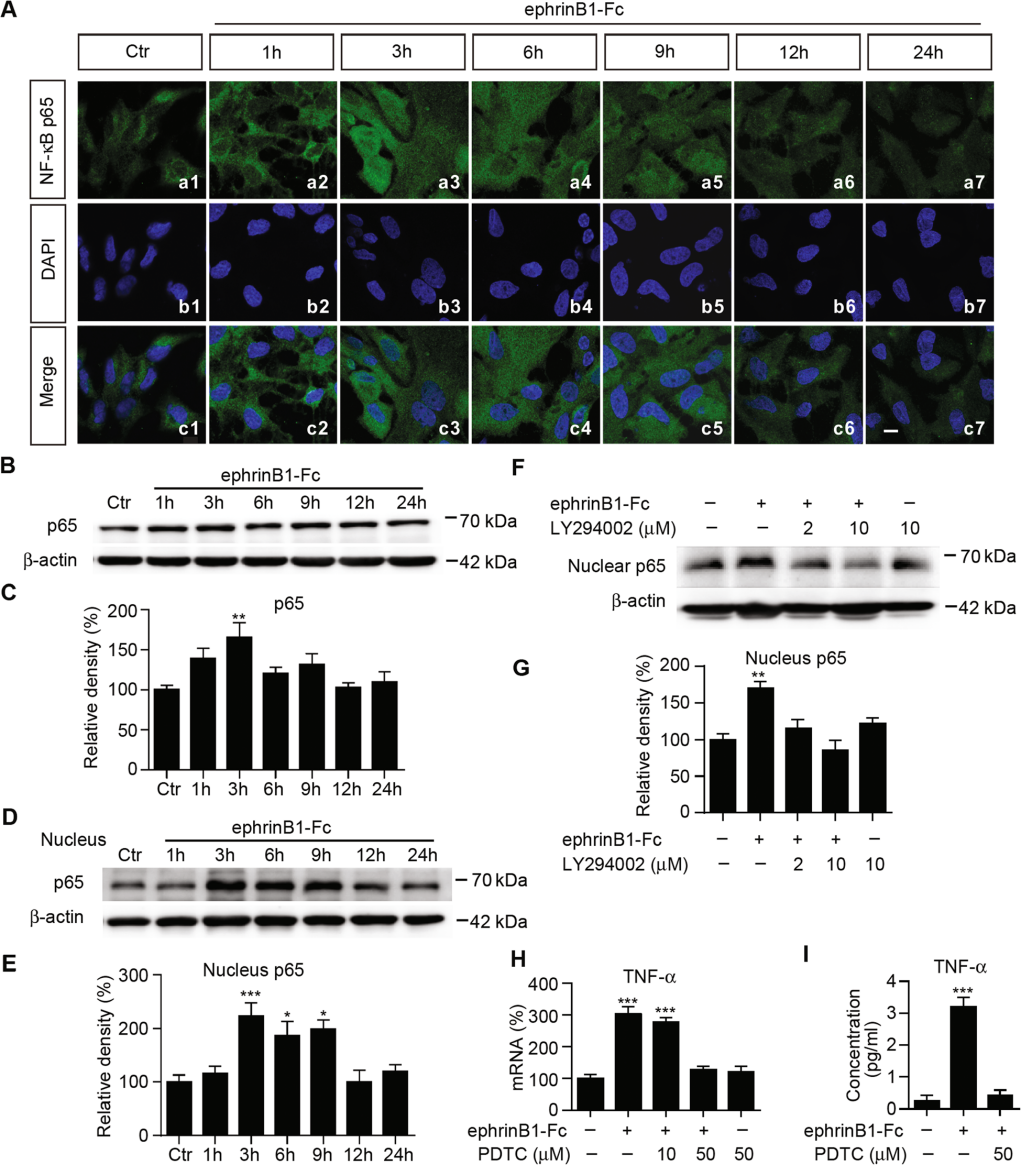
Activation of ephrinB/EphB forward signaling induces translocation of NF-κB p65 from the cytoplasm into the nucleus. **a**, Confocal laser microphotographs of cultured Müller cells, stained with the antibody against NF-κB p65 (green), showing the changes in NF-κB p65 protein expression in IgG-Fc treatment (Ctr) and ephrinB-Fc-treatment for different periods of time (a1-a7). b1-b7 are DAPI images. c1-c7 are merged images. Scale bar: 10 μm, for all the images. **b**, Representative immunoblots showing the changes in NF-κB p65 protein levels in whole cell extracts obtained from IgG-Fc-treated cells (Ctr) and those treated with ephrinB1-Fc for different periods of time. **c**, Bar chart summarizing the average densitometric quantification of immunoreactive bands of NF-κB p65 in Müller cells treated with ephrinB1-Fc for different periods of time. n = 5 for all groups. ** *P* < 0.01 vs. Ctr. **d**, Representative immunoblots showing the changes of NF-κB p65 in nucleus component of Müller cells treated with IgG-Fc (Ctr), and with ephrinB1-Fc for different periods of time. **e**, Bar chart summarizing the average densitometric quantification of immunoreactive bands of nucleus NF-κB p65 under the conditions as shown in *D*. *n* = 6 for all groups. * *P* < 0.05, and *** *P* < 0.001 vs. Ctr. **f**, Representative immunoblots showing the changes in NF-κB p65 protein levels in the nucleus component of Müller cells obtained in the presence of ephrinB1-Fc, with or without in the presence of LY294002. LY294002 (2 μM/10 μM) was added to the medium 30 min before a 3 h ephrinB1-Fc treatment. **g**, Bar chart summarizing the average densitometric quantification of immunoreactive bands of nucleus NF-κB p65 under the conditions as shown in *F*. n = 4 for all groups. ** *P* < 0.01 vs. Ctr (IgG-Fc treated group). **h**, Summary data showing that pre-incubation with PDTC inhibited the ephrinB1-Fc treatment induced increase in TNF-α mRNA levels in Müller cells. PDTC (10 μM/50 μM) was added to the medium 30 min before the ephrinB1-Fc treatment. n = 4 for all groups. **i**, Summary data showing that PDTC blocked the ephrinB1-Fc treatment induced increase in TNF-α protein levels in Müller cells. PDTC (50 μM) was added to the medium 30 min before the ephrinB1-Fc treatment. n = 4 for all groups. ** *P* < 0.01 and *** *P* < 0.001 vs. Ctr (IgG-Fc treated group)

We further explored whether the transcription factor nuclear factor-kappa B (NF-κB), a direct downstream molecule of activated PI3K/Akt signaling [15, 66], may be involved. In normal cultured Müller cells, faint NF-κB p65 positive fluorescence signals were located in the cytosol, and the signals were clearly increased from 1 h to 9 h after ephrinB1-Fc treatment by immunostaining (Fig. 9a). From the merged images it was clear that more NF-κB p65 positive signals were detected in the nucleus from 3 h to 9 h, suggesting a translocation from the cytosol to the nucleus. Changes in NF-κB p65 expression were confirmed by Western blotting (Fig. 9b-e). In the whole lysates of Müller cells, a significant increase in NF-κB p65 protein level was seen only at 3 h during a 24 h ephrinB1-Fc treatment. In the nucleus fraction, however, the relative density of NF-κB p65 was increased at 3 h and remained at this higher level for at least 6 h before returning to the control level at 12 h (Fig. 9d-e). Pre-incubation with the PI3K inhibitor LY294002 prevented the elevation of NF-κB p65 protein levels in the nucleus fraction of cultured Müller cells induced by ephrinB1-Fc treatment for 3 h (Fig. 9f-g).

Pharmacological suppression of NF-κB may attenuate the ephrinB1-Fc-induced changes in TNF-α mRNA and protein levels in cultured Müller cells. When Müller cells were pre-incubated with PDTC, a selective NF-κB inhibitor, ephrinB1-Fc no longer induced the increase in TNF-α mRNA level. Similarly, PDTC completely inhibited the ephrinB1-Fc treatment-induced elevation of TNF-α protein concentrations (Fig. 9h-i).

## Discussion

### Activation of ephrinB/EphB forward signaling in COH retinas

In the present work we first showed that the p-EphB levels in retinal extracts of both COH and ephrinB1-Fc injected eyes were significantly increased as determined by Western blotting (Fig. 2a, i). Because phosphorylation of EphB increases the enzymatic activity of EphB, increased p-EphB is often regarded as a sign of EphB activation. Moreover, ephrinB1-Fc treatment did increase p-EphB levels in purified cultured Müller cells. These results suggest an activation of ephrinB/EphB forward signaling between RGCs and Müller cells in COH retinas. It is noteworthy that EphB1 levels were increased in COH retinas, but not in EphrinB1-Fc-injected retinas. A possible explanation for this difference is that elevation of IOP may activate signaling pathways, other than ephrinB/EphB forward signaling, which induce an increase in EphB1 expression. Furthermore, we demonstrated that RGC apoptosis in COH retinas could be reduced by PP2, suggesting that activation of ephrinB/EphB forward signaling may be an important factor responsible for RGC apoptosis in glaucoma. It should be emphasized that the activation of ephrinB/EphB forward signaling did not induce Müller cell gliosis, as suggested by the unchanged GFAP expression and morphology of Müller cells following ephrinB1-Fc treatment. It means that the increase in p-EphB level may be not related to Müller cell gliosis occurring in COH retinas.

### Activation of ephrinB/EphB forward signaling promotes RGC apoptosis by up-regulating TNF-α

We further demonstrated that activation of ephrinB/EphB forward signaling in Müller cells induced by intravitreal injection of ephrinB1-Fc largely up-regulated the production of TNF-α, thereby promoting RGC apoptosis, but without inducing Müller cell gliosis. To our best knowledges, this is the first report about the up-regulated TNF-α production by the activation of ephrinB/EphB forward signaling. It has been reported that TNF-α at a concentration of picogram level may induce neuronal cell death through the silencing of survival signals [64]. Indeed, soluble TNF-α, by binding TNF receptor 1, may mediate RGC apoptosis through modulating GluA2-lacking AMPA receptors in glaucomatous eyes [13]. In the present work we also demonstrated that RGC apoptosis in COH retinas was largely reduced when TNF-α was inhibited by XPro1595. All these results suggest that activation of ephrinB1/EphB1 forward signaling induced TNF-α production may play an important role in RGC apoptosis in glaucomatous eyes. We previously showed that EphB/ephrinB reverse signaling was also activated in COH retinas, which contributes to RGC apoptosis through decreasing the Ca^2+^-impermeable GluA2-containing AMPA receptor expression in the membrane of RGCs [16]. It is of interest to note that in the ONH of spontaneous glaucomatous DBA/2 J mice, the activation of EphB/ephrinB reverse signaling may contribute to the axon loss by causing an elevation of intracellular Ca^2+^ concentrations in RGC axons [17, 21], while ephrinB/EphB forward signaling may play a protective role for RGC axons, as suggested by the experiment showing that more serious degeneration of RGC axons was seen in experimental glaucomatous EphB2^−/−^ and EphB3^−/−^ mice [19]. Moreover, it was reported that in the rat forebrain traumatic injury activation of ephrinB3/EphB3 forward signaling attenuated neuronal death [63]. In this context it seems quite unique in the retinas with COH that the activation of both reverse and forward signaling of the ephrinB/EphB system, with EphB and ephrinB existing in a glial element and a neuronal element respectively, contributes to RGC apoptosis through different pathways.

It is known that production of TNF-α in glaucomatous retinas may be resulted by Müller cell gliosis [13, 41, 61, 62]. It has been shown that TNF-α released from the activated Müller cells may be mediated by p38-MAPK signaling pathway [5, 41, 61]. Indeed, we previously showed that this signaling pathway was also involved into mGluR I activation induced Müller cell gliosis [23]. In other words, TNF-α production from Müller cells in COH retinas could be due to ephrinB/EphB forward signaling activation and/or gliosis of Müller cells. A speculation about how these two mechanisms may work in COH retinas may be proposed as follows. Since the increase in EphB1 expression in Müller cells and ephrinB2 in RGCs was observed as early as 1 day (G1d) after IOP elevation [16], but a significant increase of GFAP expression was only observed on G1w in COH retinas [33], it is reasonable to assume that at a very early phase of IOP elevation it may be the activation of ephrinB/EphB forward signaling that triggers the production of TNF-α from Müller cells. Steady IOP elevation could cause Müller cell gliosis, also resulting in the production of TNF-α and other inflammatory factors at a late phase. During this phase the glutamate concentration in the extracellular space is steadily increased, which could lead to more Müller cells to be reactivated and make ephrinB/EphB forward signaling activation stronger by stimulating mGluR I [33] and NMDA receptors (this work) respectively. In short, the two mechanisms may work in concert in the time domain to aggravate RGC damage. It seems likely that suppression of ephrinB/EphB forward signaling may be regarded as a new strategy for ameliorating RGC apoptosis in glaucoma.

### Involvement of NMDA receptors in ephrinB/EphB forward signaling activation induced TNF-α production in Müller cells

In glaucoma extracellular glutamate levels in the retina are elevated, which could be caused by reduced expression of glutamate transporters [28, 42, 65]. Since glutamate fails to increase obvious Ca^2+^ transition in Müller cells in rats and guinea pigs, it is generally thought that NMDA receptors are not involved in Müller cell gliosis [7, 45, 46]. Indeed, the activation of ephrinB/EphB forward signaling caused an increase in p-NR2B subuint expression in Müller cells, but did not change GFAP expression, a sign of Müller cell gliosis. While NMDA receptors do not contribute to Müller cell gliosis, our results strongly suggest that these receptors may be involved in the TNF-α production due to ephrinB/EphB forward signaling activation. This suggestion is supported by the result that the elevation of TNF-α mRNA and protein levels in Müller cells due to ephrinB1-Fc treatment was blocked by the selective NR2B antagonist RO25–6981 (Fig. 7). Previous studies have demonstrated that the effects of activation of ephrinB/EphB bi-directional signaling on neuronal functions were mediated through modulating NMDA receptors. For example, EphB may interact directly with NMDA receptors, thereby modulating central synaptic functions by changing NMDA receptor-dependent Ca^2+^ influx through increasing tyrosine phosphorylation of NR2B [8, 14, 30, 47, 50, 56, 60]. Additionally, intrathecal administration of ephrinB2-Fc to activate ephrinB/EphB forward signaling in adult rats could induce behavioral thermal hyperalgesia, which was mediated by NR2B tyrosine phosphorylation [56].

Overall, the PI3K/Akt/NF-κB signaling pathway, being responsible for the production of TNF-α, is not much different from those mediating the actions on neurons following the activation of NR2B [4, 9, 36, 40, 52, 68]. However, there are two points that should be emphasized. First, prevention of the elevated expression of PI3K (Fig. 8) and the production of TNF-α (Fig. 7) following the activation of EphB receptors by RO25–6981 suggests a direct activation of PI3K/Akt in Müller cells by the Ca^2+^ influx through NR2B subunit, similar to that observed in cortical and hippocampal neurons [4, 9, 36, 52, 68], but different from an indirect action of Ca^2+^, observed in rat cortical neurons, on PI3K/Akt through the adaptor protein APPL1, which was mediated by the synaptic NMDA receptor activity [67]. Second, it has been well established that activated Akt enables the NF-κB dimers to enter the nucleus and activating specific target genes that are responsible for inflammation and immune response [15, 26, 31]. This notion is supported by our result, showing that activated ephrinB/EphB forward signaling induced a significant increase in NF-κB p65 expression in the nucleus component of Müller cells in mediation of p-Akt. This is also consistent with a previous work showing that NMDA treatment induced NF-κB activation in Müller cells, and then increased TNF-α production [40].

## Conclusions

**Fig. 10:**
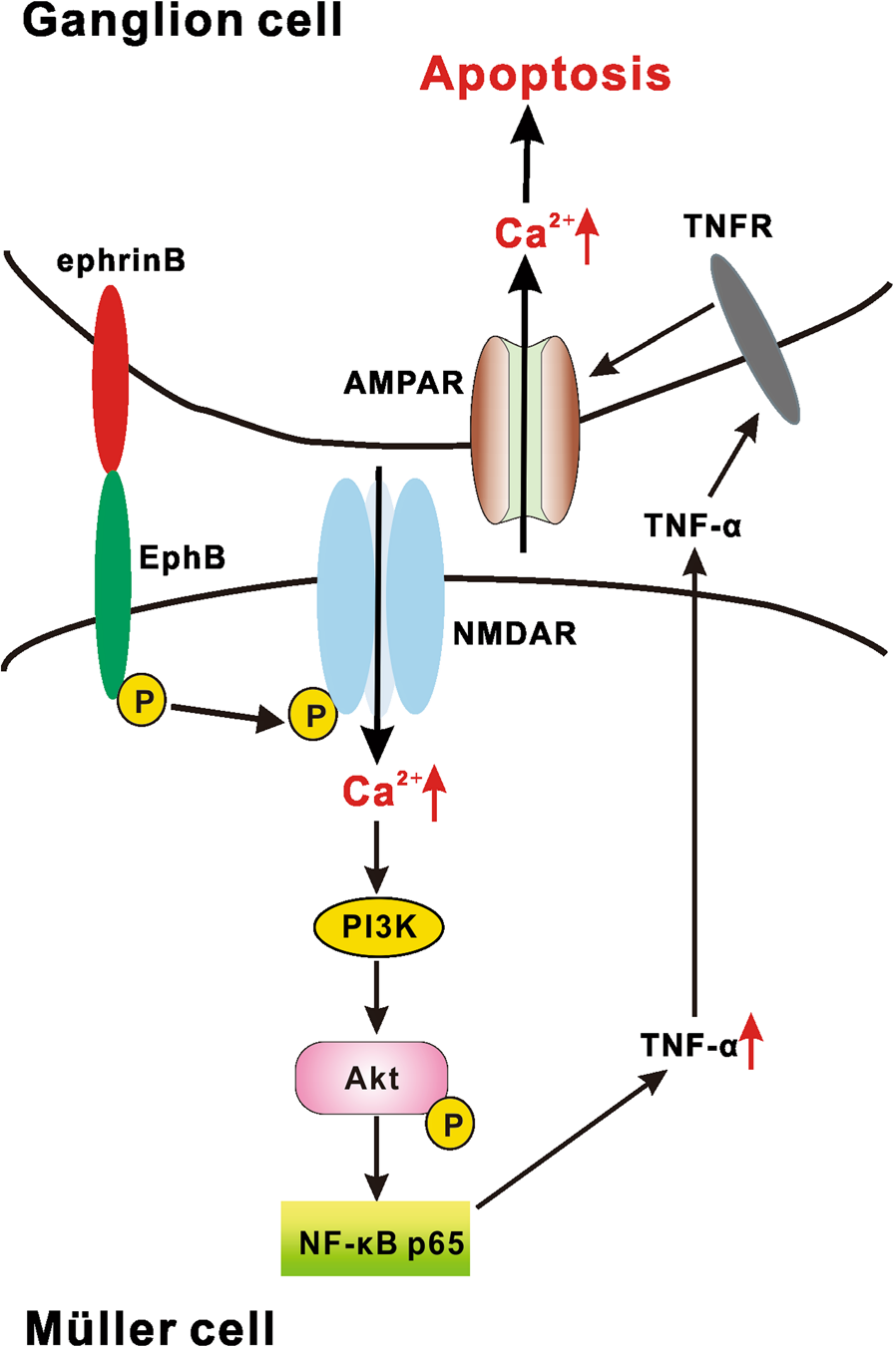
Schematic diagram showing the signaling pathway involved in ephrinB/EphB forward signaling activation-induced TNF-α production in Müller cells and RGC apoptosis in COH retinas. AMPAR: AMPA receptor; NF-κB: nuclear factor-kappa B; NMDAR: NMDA receptor; PI3K: phosphatidylinositol 3-kinases; TNF-α: tumor necrosis factor-α; TNFR: TNF receptor

In the present work, we demonstrate that ephrinB/EphB forward signaling is activated in Müller cells in a chronic ocular hypertension model, which up-regulates TNF-α production via a distinct NR2B/PI3K/Akt/NF-κB signaling pathway and contributes to RGC apoptosis (Fig. 10), but without inducing Müller cell gliosis.

## Abbreviations

AMPA: α-amino-3-hydroxy-5-methyl-4-isoxazole-propionic acid
COH: Chronic ocular hypertension
ephB: Erythropoietin-producing hepatocyte receptor
GFAP: Glial fibrillary acidic protein
GluA2: Glutamate receptor subunit A2
NF-κB: Nuclear factor-kappa B
NR2B: *N*-methyl-*D*-aspartate receptor subunit 2B
ONH: Optic nerve head
PI3K: Phosphatidylinositol 3-kinases
RGC: Retinal ganglion cell
TNF-α: Tumor necrosis factor-α
TUNEL: Terminal dUTP nick end labeling
